# Lecanemab treatment for Alzheimer's Disease of varying severities and associated serum biomarkers monitoring: a real‐world study

**DOI:** 10.1002/alz70856_101739

**Published:** 2025-12-25

**Authors:** Xueping Chen

**Affiliations:** ^1^ West China Hospital, Chengdu, Sicuan, China; West China Hospital, Sichuan University, Chengdu, Sichuan, China

## Abstract

**Background:**

Alzheimer's disease (AD) is a progressive neurodegenerative disorder and the leading cause of dementia, posing significant global health and societal challenges. Lecanemab is a disease‐modifying therapy approved for mild AD. In this report, we present real‐world data on the efficacy and safety of lecanemab, and explore the role of AD‐related serum biomarkers in monitoring treatment efficacy in a real‐world setting.

**Method:**

Cognitive function was assessed using AD assessment scale‐cognition (ADAS‐Cog), clinical dementia rating scale—sum of boxes (CDR‐SB), montreal cognitive assessment (MoCA), mini‐mental state examination (MMSE), and frontal assessment battery (FAB) at baseline and every follow‐up. Serum biomarkers, including neurofilament light chain (NFL), glial fibrillary acidic protein (GFAP), Aβ 1‐40, Aβ 1‐42, *p*‐tau 181, and *p*‐tau 217, were monitored with chemiluminescence assay at all time points.

**Result:**

Fifty‐one AD patients meeting ATN criteria were enrolled (CDR 0.5: *n* = 25; CDR 1: *n* = 14; CDR 2: *n* = 12). Significant improvements were observed in ADAS‐Cog (+4.36 points, *p* <0.001), CDR‐SB (+0.31 points, *p* = 0.010), and MoCA (+1.40 points, *p* = 0.002) at 7 months, with no significant change in MMSE. Only serum *p*‐tau 181 decreased significantly (*p* = 0.031), while *p*‐tau 217 showed a non‐significant trend. Spearman correlation analysis revealed associations between serum *p*‐tau181, *p*‐tau217, and cognitive scores. The adjusted linear mixed‐effects model indicated a significant association between serum *p*‐tau 181 and ADAS‐Cog scores (β=0.3546, *p* <0.001). No serious adverse events occurred. Infusion reactions were reported in 7.69% of patients, and 9.62% discontinued due to asymptomatic amyloid‐related imaging abnormalities (ARIA).

**Conclusion:**

In the real world, lecanemab may be safe and effective in the treatment of mild‐to‐moderate AD, and dynamic monitoring of serum *p*‐tau 181 may be helpful to observe the efficacy during treatment. AD patients with severe cognitive impairment and significant white matter lesions should be closely monitored for adverse reactions, such as ARIA.

